# The Soluble Factor from Oral Cancer Cell Lines Inhibits Interferon-γ Production by OK-432 via the CD40/CD40 Ligand Pathway

**DOI:** 10.3390/cancers13133301

**Published:** 2021-06-30

**Authors:** Go Ohe, Yasusei Kudo, Kumiko Kamada, Yasuhiro Mouri, Natsumi Takamaru, Keiko Kudoh, Naito Kurio, Youji Miyamoto

**Affiliations:** 1 Department of Oral Surgery, Tokushima University Graduate School, 3-18-15 Kuramoto-cho, Tokushima 770-8504, Japan; kamakama@tokushima-u.ac.jp (K.K.); takamaru@tokushima-u.ac.jp (N.T.); kkudoh@tokushima-u.ac.jp (K.K.); kurio.naito@tokushima-u.ac.jp (N.K.); miyamoto@tokushima-u.ac.jp (Y.M.); 2 Dentistry and Oral Surgery, Takamatsu Municipal Hospital, 847-1 Ko Busshozan-cho, Takamatsu 761-8538, Japan; 3 Department of Oral Bioscience, Tokushima University Graduate School, 3-18-15 Kuramoto-cho, Tokushima 770-8504, Japan; yasusei@tokushima-u.ac.jp (Y.K.); ymouri@tokushima-u.ac.jp (Y.M.)

**Keywords:** OK-432, conditioned medium, interferon-γ, oral cancer, CD40

## Abstract

**Simple Summary:**

OK-432 is a potent immunotherapy agent for several types of cancer, including oral cancer. We previously reported that OK-432 treatment can induce the production of high levels of IFN-γ from peripheral blood mononuclear cells (PBMCs). Moreover, the IFN-γ production from PBMCs by OK-432 is impaired by conditioned media (CM) from oral cancer cells. To determine the inhibitory mechanism of IFN-γ production by CM, the genes involved in IFN-γ production was retrieved by cDNA microarray analysis. We found that CD40 played a key role in IFN-γ production via IL-12 production. Although the expression levels of CD40 were upregulated by OK-432 treatment in PBMCs, CM inhibited OK-432-induced CD40 expression. These findings suggest that uncertain soluble factor(s) in CM may suppress IFN-γ production via the CD40/CD40L–IL-12 axis in PBMCs.

**Abstract:**

(1) Background: OK-432 is a penicillin-killed, lyophilized formulation of a low-toxicity strain (Su) of *Streptococcus pyogenes* (Group A). It is a potent immunotherapy agent for several types of cancer, including oral cancer. We previously showed that (i) OK-432 treatment induces a high amount of IFN-γ production from peripheral blood mononuclear cells (PBMCs), and (ii) conditioned medium (CM) from oral cancer cells suppresses both the IFN-γ production and cytotoxic activity of PBMCs driven by OK-432. The aim of this study was to determine the inhibitory mechanism of OK-432-induced IFN-γ production from PBMCs by CM. (2) Methods: We performed cDNA microarray analysis, quantitative RT-PCR, and ELISA to reveal the inhibitory mechanism of CM. (3) Results: We found that CD40 plays a key role in IFN-γ production via IL-12 production. Although OK-432 treatment upregulated the expression levels of the *IL-12p40*, *p35*, and *CD40* genes, CM from oral cancer cells downregulate these genes. The amount of IFN-γ production by OK-432 treatment was decreased by an anti-CD40 neutralizing antibody. (4) Conclusions: Our study suggests that uncertain soluble factor(s) produced from oral cancer cells may inhibit IFN-γ production from PBMCs via suppressing the CD40/CD40L–IL-12 axis.

## 1. Introduction

OK-432 (Chugai Pharmaceutical, Tokyo, Japan) is a penicillin-killed, lyophilized formulation of a low-toxicity strain (Su) of *Streptococcus pyogenes* (Group A). It is known as a potent immunotherapy agent for several types of cancer, including oral cancer [1,2,3,4,5]. Administration of OK-432 induces cytotoxic T lymphocytes (CTL) and cytotoxic macrophages and activates anti-tumor effecter cells, including lymphokine-activated killer (LAK) cells and natural killer (NK) cells [6]. Moreover, OK-432 induces the production of anti-tumor cytokines, such as interleukin (IL)-2, IL-12, interferon (IFN)-γ, and tumor necrosis factor (TNF)-α from Th1 cells, NK cells, and monocytes/macrophages. Thus, OK-432 shows an anti-tumor effect against several types of cancer via activation of immune cells [7,8,9,10]. OK-432 also induces cancer antigen-specific CTL via maturation of antigen-presenting cells [11]. Our previous study showed that the combination therapy of OK-432 with radiation and UFT (an oral fluoropyrimidine formulation with the combination of tegafur and uracil at a 1:4 ratio; Taiho Pharmaceutical Co., Tokyo, Japan) exhibits a potent anti-tumor effect against oral cancer [12,13].

CD40 is a regulatory molecule of IL-12. CD40 is a membrane antigen belonging to the TNF receptor family and is mainly expressed on B cells [14]. CD40 is also expressed on antigen-presenting cells, such as dendritic cells and macrophages [15]. Interaction between CD40 on antigen-presenting cells and CD40L on activated T cells induces IL-12p40 expression. IL-12 is known to activate NK cells [16,17,18]. A glycoprotein, p70, forms a heterodimer consisting of p40 and p35 subunits and functions as an IL-12 (active form p70) [19,20]. In general, p35 is produced constantly by B cells, macrophages, and dendritic cells [21], whereas p40 is produced by these cells upon antigen stimulation. IL-12p40 is a potent inducer of IFN-γ via promoting T cell differentiation and activation [22]. IFN-γ is the only member of the type II class of interferons and secreted mainly from activated T cells, antigen-presenting cells, and NK cells. IFN-γ induces cytotoxic T cells, activates NK cells, enhances anti-tumor effect of macrophages, and enhances the ability of tumors to present MHC Class I and MHC Class II antigens, and thus is known as representative anti-tumor cytokines [23].

We have previously shown that the serum concentration of IFN-γ in patients with advanced cancer treated by OK-432 is lower than in patients with early cancer treated with OK-432 [24]. This finding suggests that the anti-tumor response of OK-432 in patients with oral cancer may be suppressed by cancer progression. We hypothesized that certain soluble factors secreted from cancer cells may decrease the effect of OK-432 on IFN-γ production from peripheral blood mononuclear cells (PBMCs). To demonstrate this hypothesis, we established an in vitro simulation model. We simulated the immune status in oral cancer patients by the addition of conditioned media (CM) derived from oral cancer cells to PBMCs derived from healthy volunteers [25]. Then, we demonstrated that CM containing unknown soluble factor(s) decreased the IFN-γ production from PBMCs by OK-432 treatment. We also found that representative immunosuppressive cytokines including IL-4, IL-6, IL-10, TGF (transforming growth factor)-β, and VEGF (vascular endothelial growth factor) were not contained in the CM from oral cancer cells [25]. To identify an unknown molecule(s) could be key to developing an effective immunotherapy against oral cancer. Therefore, herein, we investigated the inhibitory mechanism of CM against OK-432-induced IFN-γ production from PBMCs.

## 2. Materials and Methods

### 2.1. Cells and Cell Culture

Both B88 [26] and HNt cells [27] were from a human oral squamous cell carcinoma cell line. The TYS cells [28] were from a human oral adenoid squamous carcinoma cell line. All cells were cultured in Dulbecco’s Modified Eagle’s Medium (DMEM) (Sigma-Aldrich, St. Louis, MO, USA, D5796-500ML) supplemented with 10% (*V/V*) fetal bovine serum (FBS) (Premium FBS, Bio-Whittaker, Walkersville, MD, USA, BW14-501F), 100 µg/mL of streptomycin, and 100 U/mL of penicillin (Sigma-Aldrich, P4333-100ML). The PBMCs were cultured in RPMI 1640 (Sigma-Aldrich, R8758-500ML) supplemented with 10% FBS. All of the cells were incubated in a humidified atmosphere of 95% air and 5% CO_2_ at 37 °C.

### 2.2. Preparation of PBMCs

The PBMCs from a healthy volunteer were obtained by Ficoll–Hypaque gradient density centrifugation according to Boyüm’s protocol [29].

### 2.3. Preparation of CM

The sub-confluent of oral cancer cells (B88, HNt and TYS cells; 2.5 × 10^6^ cells) in 100 mm dishes (Falcon 100-mm TC-treated Cell Culture Dish, CORNING, Glendale, AZ, USA, 353003) in complete culture medium was subjected to CM collection. The cells were washed three times by phosphate-buffered saline (−), and then cells were cultured for an additional 72 h in 10 mL of the serum-free media. The media were filtrated using a 0.2 µm pore membrane (Corning 28 mm Diameter Syringe Filters, 0.2 µm Pore PES Membrane, CORNING, 431229). Then, they were used for the experiments as CM.

### 2.4. In Vitro Simulation Model of the Oral Cancer Patients

Establishment of an in vitro simulation model of patients with oral cancer was previously reported [25]. Briefly, PBMCs (1 × 10^6^ cells/mL) were maintained in RPMI 1640 supplemented with 10% FBS. The media were replaced with CM-containing media at the CM volume of 1/8, 1/4, or 1/2. Then, FBS and OK-432 were added to the final concentrations of 10% and 1 µg/mL, respectively.

### 2.5. Isolation of Total RNA

The PBMCs were cultured in RPMI 1640 medium supplemented with 10% FBS with or without OK-432 (1 µg/mL) and CM was retrieved. Then, the total RNA was extracted using an ISOGEN RNA extraction mixture (NIPPON GENE, Toyama, Japan, 311-02501).

### 2.6. cDNA Microarray

cDNA from untreated PBMCs, PBMCs treated with OK-432 (1 µg/mL) for 12 h, or PBMCs treated with OK-432 (1 µg/mL) followed by CM (at volume of 1/2) derived from B88 cells were subjected to a cDNA microarray (GENECOM, Ehime, Japan). The cDNA microarray was equipped with 29,098 total human genes. Gene Ontology (GO) analysis was performed for genes whose expression was upregulated more than 2-fold and downregulated less than one-half by OK-432/CM treatment compared to OK-432 treatment using Metascape. Then, a heatmap was created for the selected cytokine genes using the R package “pheatmap.”

### 2.7. Quantitative Reverse Transcription–Polymerase Chain Reaction

Five micrograms of total RNA was reverse-transcribed by Moloney murine leukemia virus reverse transcriptase (Promega, Madison, WI, USA, M170A) at 42 °C for 60 min in a 20 µL mixture with a random primer (Promega, C1181). Two microliters of the reverse-transcribed mixture was subjected to quantitative PCR for *IFN-γ* (Hs00174143), *IL-12p40* (Hs00233688), *IL-12p35* (Hs00168405), *CD40* (Hs00154345), *CD40 Ligand* (*CD40L*) (Hs00163934), and *GAPDH* mRNA expression. The expression levels were measured by a TaqMan^®^ gene expression assay (Applied Biosystems, Foster City, CA, USA) and an ABI PRISM7000 sequence detection system (Applied Biosystems).

### 2.8. Enzyme-Linked Immunosorbent Assay (ELISA)

The concentrations of cytokines (IFN-γ and IL-12) in the cell culture supernatant were measured with an ELISA kit according to the manufacturer’s instructions (Quantikine ELISA, Minneapolis, MN, USA, DIF50C and D1200). All experiments were performed three times independently, and we showed the representative data.

### 2.9. Effects of Anti-IL-12 Neutralizing Antibodies (Abs), Recombinant IL-12 (rIL-12), or Anti-CD40 Ab on PBMCs by OK-432 Treatment

The PBMCs were treated with the following neutralizing Abs or rIL-12: An anti-IL-12 Ab (clone 3007; 0.1–10 µg/mL), an anti-CD40 Ab (clone 6708; 10–100 pg/mL), or a rIL-12 (clone 25209; 0.1–100 ng/mL). The cells were treated with OK-432 for 24 h; then, the IFN-γ level in the culture supernatant was measured. All neutralizing Abs and rIL-12 used were purchased from R&D Systems (Minneapolis, MN, USA).

### 2.10. Data Analysis

RNA-sequencing data from 515 HNSCC samples were released by TCGA PanCancer Atlas. The RNASeqV2 data from TCGA were analyzed using cBioPortal (http://cbioportal.org, Version 3.6.18 (accessed on 20 May 2021)). For investigating the prognostic value of the target genes (CD40/CD40L, IL12, and IFN-γ) in HNSCC cases, we divided the cases into two groups, “low” and “high,” based on the median expression level of each gene. Then, we compared the survival rate of the “low” group with the “high” group by a log-rank test.

### 2.11. Statistical Analysis

The obtained data are expressed as the mean ± standard deviation using analysis of variance (ANOVA) with *p* < 0.05 as the level of statistical significance. All statistical analyses were conducted using Prism (GraphPad Software, San Diego, CA, USA).

## 3. Results

### 3.1. Effect of CM on the Cytokine Gene Expressions in PBMCs by OK-432 Treatment (cDNA Microarray)

**Figure 1:**
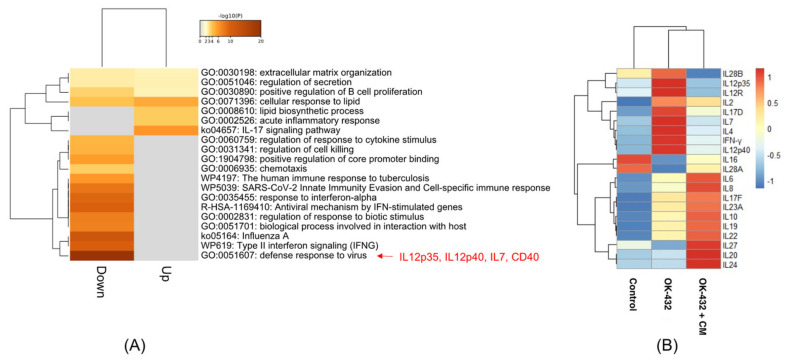
Expression of cytokine genes detected by cDNA microarray. (**A**) Heatmap of GO-enriched terms. GO analysis was performed for genes whose expression was upregulated more than 2-fold and downregulated less than one-half by OK-432/CM treatment compared to OK-432 treatment using Metascape. (**B**) cDNA microarray analysis revealed that some cytokines were upregulated by OK-432 treatment and suppressed by OK-432 treatment with CM from B88 cells in PBMCs. The expression values are centered and scaled in the row direction.

To identify the molecules in CM that reduce the IFN-γ production from PBMCs by OK-432 treatment, we compared the gene expression profiles among the control, OK-432 treatment, and OK-432 treatment with the CM from B88 cells by microarray. We previously examined the inhibitory effect of the CM from B88, HNt, and TYS cells on IFN-γ production from PBMCs by OK-432 treatment [25]. The CM from B88 inhibited the IFN-γ production from PBMCs by OK-432 treatment more than the CM from HNt and TYS. In this study, therefore, we used the CM from B88 cells to inhibit IFN-γ production. GO enrichment analysis revealed that inflammation-related GOs, including *IL-12p35, IL-12p40*, and *CD40*, were enriched in the genes whose expression was downregulated by CM treatment (Figure 1A). The most enriched GO term in the downregulated genes by CM treatment was “defense response to virus.” Interestingly, the IFN-γ-related GO terms were significantly enriched. In the most enriched GO term, the *IL-12p35*, *IL-12p40*, and *CD40* genes were included. Interestingly, *IL-12p35*, *IL-12p40*, and other cytokines (*IL-7*, *IL-12R*, *IL-2*, *IL-17D*, and *IL-4*), as well as *IFN-γ*, were upregulated by OK-432 treatment and were downregulated by OK-432 treatment with CM derived from B88 cells in PBMCs (Figure 1B). In this study, we focused on IL-12-related genes, *IL-12p35* and *IL-12p40*, in the subsequent experiments.

### 3.2. Blocking the Effect of CM on the mRNA Expression of IL-12-Related Molecules, IL-12p40, and IL-12p35 in PBMCs by OK-432 Treatment

**Figure 2:**
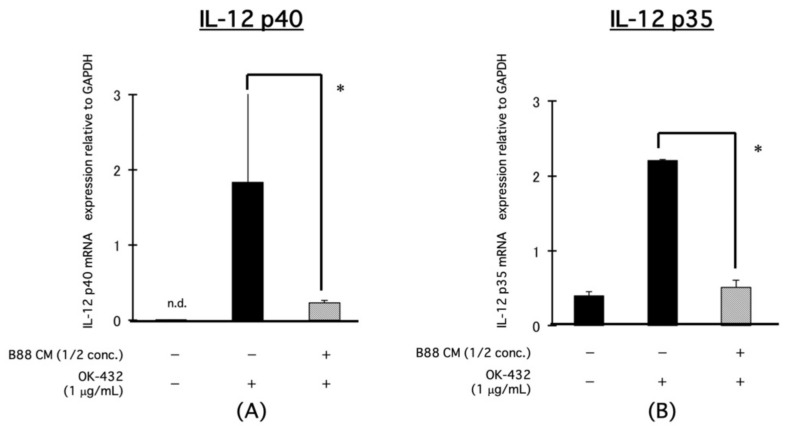
Blocking the effect of CM on the mRNA expression of IL-12-related molecules, IL-12p40, and IL-12p35 on PBMCs induced by OK-432 treatment. In the PBMCs, OK-432 was treated for 24 h with or without CM. Then, the mRNA expression of IL-12p40 and IL-12p35 was analyzed by real-time PCR. All samples were analyzed in triplicate. (**A**) IL-12p40 and (**B**) IL-12p35. * *p* < 0.001; n.d., not detectable.

The expression levels of IL-12p40 and IL-12p35 mRNA in PBMCs were increased by treatment with OK-432 and were significantly decreased in the presence of CM derived from B88 cells by real-time PCR (Figure 2A,B).

### 3.3. Blocking the Effect of CM on the mRNA Expression of IL-12-Related Molecules, IL-12p40, and IL-12p35 in PBMCs by OK-432 Treatment

**Figure 3:**
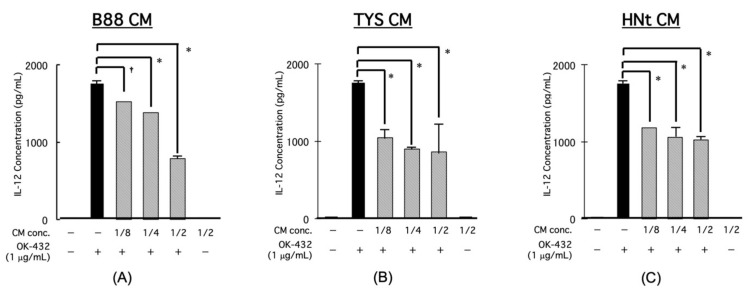
Effect of CM from oral cancer cells on IL-12 production from PBMCs by OK-432 treatment. The PBMCs were treated by OK-432 for 24 h with various concentrations of CM. Then, the amount of IL-12 in the culture supernatant was measured by ELISA. All samples were analyzed in triplicate. (**A**) B88 cells, (**B**) TYS cells, and (**C**) HNt cells. * *p* < 0.001; † *p* < 0.05.

We previously showed that IFN-γ production from PBMCs by OK-432 treatment can be decreased by the CM from oral cancer cells in a concentration-dependent manner [15]. The IL-12 production from the PBMCs was significantly increased by OK-432 treatment and was decreased by adding CM from different oral cancer cells (B88, TYS, and HNt cells) in a concentration-dependent manner (Figure 3A–C).

### 3.4. Effects of Anti-IL-12 Neutralizing Ab or rIL-12 on IFN-γ Production from PBMCs by OK-432 Treatment

**Figure 4:**
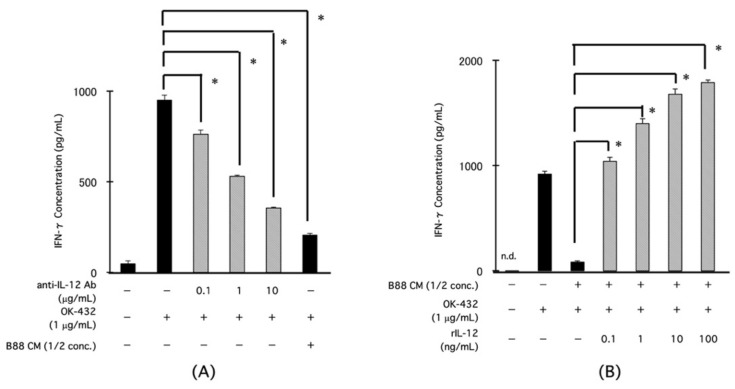
Effect of an anti-IL-12 neutralizing Ab or a rIL-12 protein on IFN-γ production from PBMCs by OK-432 treatment. The PBMCs were treated with OK-432 for 24 h with or without an anti-IL-12 neutralizing Ab. Then, the amount of IFN-γ was measured by ELISA (**A**). In the presence of CM, the PBMCs were treated by OK-432 with or without a rIL-12 protein for 24 h. Then, the amount of IFN-γ was measured by ELISA (**B**). All samples were analyzed in triplicate. * *p* < 0.001; n.d., not detectable.

To investigate the effect of IL-12 on IFN-γ production from PBMCs by OK-432 treatment, we examined the IFN-γ production after treatment with an anti-IL-12 neutralizing Ab or a recombinant IL-12 (rIL-12) protein. The IFN-γ production from the PBMCs was decreased by anti-IL-12 neutralizing Ab treatment in a concentration-dependent manner (Figure 4A). On the contrary, the CM-mediated inhibition of IFN-γ production was rescued by treatment with an rIL-12 protein in a concentration-dependent manner (Figure 4B).

### 3.5. Blocking Effect of CM on the mRNA Expression of IL-12-Related Molecules, CD40, and CD40L in PBMCs by OK-432 Treatment

**Figure 5:**
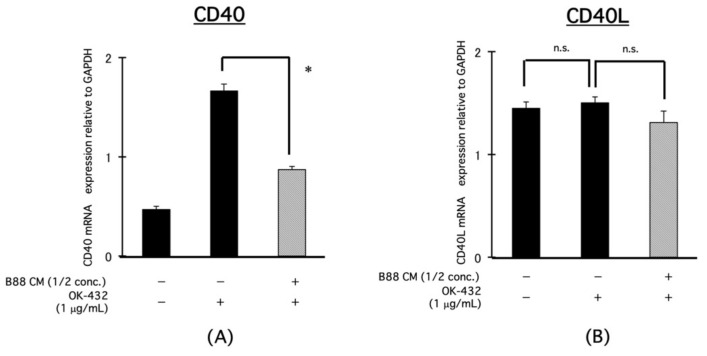
Blocking effect of CM on the mRNA expression of IL-12-related molecules, CD40, and CD40L on PBMCs by OK-432 treatment. The PBMCs were treated with OK-432 for 24 h with or without CM. Then, the mRNA expression of CD40 and CD40L was analyzed by real-time PCR. All samples were analyzed in triplicate. (**A**) CD40 and (**B**) CD40L. * *p* < 0.001; n.s., not significant.

**Figure 6:**
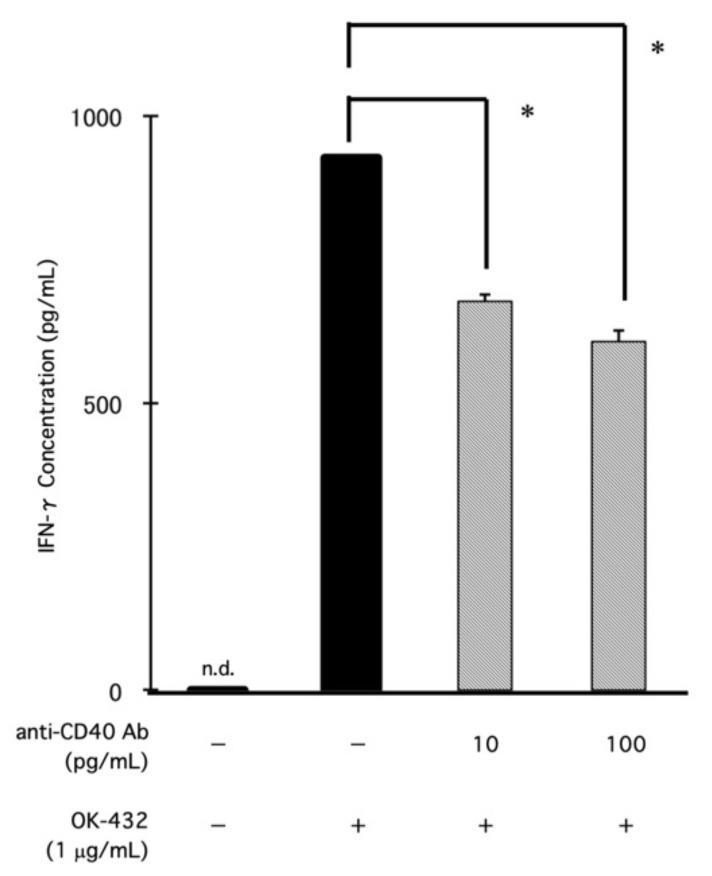
Blockage effect of an anti-CD40 neutralizing Ab on IFN-γ production from PBMCs by OK-432 treatment. The PBMCs were treated with OK-432 for 24 h with or without an anti-CD40 neutralizing Ab. Then, the amount of IFN-γ was measured by ELISA. All samples were analyzed in triplicate. * *p* < 0.001; n.d., not detectable.

We found the involvement of IL-12 in the suppression of IFN-γ production from PBMCs by OK-432 treatment in the presence of CM derived from B88 cells. Therefore, we focused on CD40 and CD40L, which are upstream of IL-12 signaling pathway. It is known that CD40L expressed on activated T cell ligates to CD40, and consequently IL-12 production is induced. Herein, the expression of CD40 mRNA in PBMCs was increased by OK-432 treatment and was significantly decreased in the presence of CM derived from B88 cells (Figure 5A). However, the CD40L mRNA expression was not altered by OK-432 treatment with or without CM (Figure 5B). Then, we examined the effect of an anti-CD40 neutralizing Ab on the IFN-γ production from PBMCs by OK-432 treatment. IFN-γ production from PBMCs by OK-432 treatment was decreased by an anti-CD40 neutralizing Ab (Figure 6).

## 4. Discussion

This study clearly demonstrated that the IL-12 pathway is important for IFN-γ production from PBMCs by OK-432 treatment. IL-12 is identified as a cytokine for activating NK cells [16,17,18] and is a 70 kDa glycoprotein that forms a heterodimer consisting of p40 and p35 subunits [19,20]. Generally, both IL-12p40 and IL-12p35 are generated in B cells and macrophages or antigen-presenting cells, such as DCs [21]. IL-12p40 promotes the differentiation and activation of T cells and then enhances the IFN-γ production from T cells [30,31]. IFN-γ production from PBMCs by OK-432 treatment was suppressed by CM and was rescued by rIL-12 treatment, suggesting a possible new therapy against oral cancer with the combination IL-12 and OK-432. However, it has been reported that large amounts of inflammatory cytokines, such as TNF-α, are induced by the administration of IL-12, and serious adverse events occur in patients [32]. Therefore, adenovirus expressing the *IL-12* gene is developed and a clinical phase study to produce IL-12 under physiological conditions is underway [33,34]. It has also been reported to be difficult to regulate the expression level of IL-12, which does not have a drastic anti-tumor effect. Based on these findings, we investigated the genes upstream of IL-12 that would enable patients to produce physiologic IL-12. We identified CD40, which regulates IL-12 expression. In this study, CD40 expression on PBMCs was increased by OK-432 treatment, and the increased CD40 expression was significantly inhibited in the presence of CM (Figure 4A,B). Furthermore, increased IFN-γ production from PBMCs by OK-432 treatment was decreased by adding anti-CD40 Ab (Figure 5). These findings suggest that certain soluble factor(s) in CM may decrease IFN-γ production via inhibiting CD40 expression on PBMCs. It is interesting to examine the combination effects of OK-432 and IL-12 or upstream of IL-12, such as CD40/CD40L, on tumor progression both in vitro and in vivo. 

We previously found that IL-10 and TGF-ß contribute to the decreased expression of CD40 [35,36]. However, we previously showed that neutralizing IL-10 and TGF- ß by using an antibody does not rescue the inhibition of IFN-γ production from PBMCs by adding CM [25], indicating that certain soluble factor(s), except for IL-10 and TGF-ß, in CM may regulate CD40 expression. To clarify the mechanism on the regulation of CD40 expression by CM, further experiments will be required.

IFN-γ production from PBMCs by adding CM may be inhibited by activation of the CD40/CD40L signaling pathway. However, it is difficult to enhance CD40L expression, because the half-life of the CD40L protein is short [37]. While anti-CD40 agonistic Ab has a similar effect on CD40L, it shows various immune responses through the maturation of DCs [38]. Interestingly, only anti-CD40 agonistic Ab treatment showed a strong anti-tumor effect by activating cytotoxic T cells in a mouse B cell lymphoma transplantation model [39]. Although now anti-CD40 agonistic Ab is not used clinically, combination therapy with OK-432 and anti-CD40 agonistic Ab may have potential in novel cancer immunotherapy.

**Figure 7:**
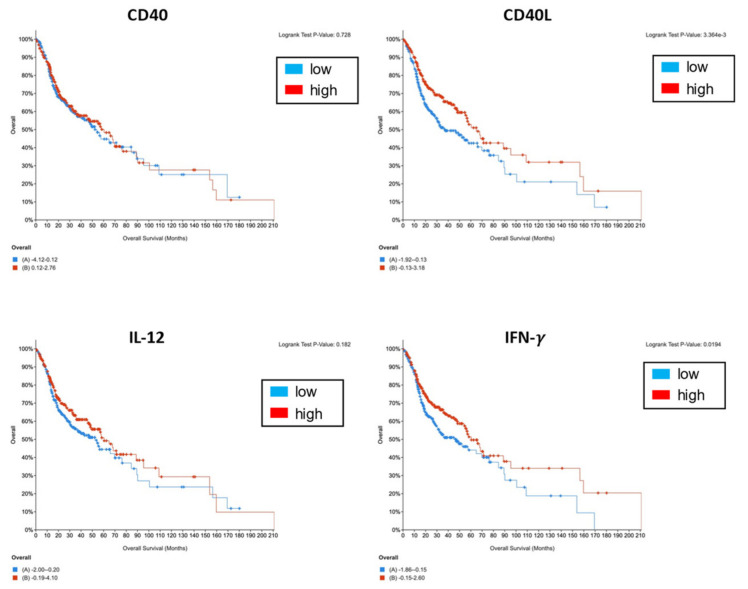
Correlation of the expression of CD40/CD40L, IL12, and IFN-γ with the prognosis of patients with head and neck squamous cell carcinoma. Patients with high expression of CD40L and IFN-γ show better overall survival. Red (high) and blue (low) show the expression of CD40/CD40L, IL12, and IFN-γ, as assessed from the TCGA data.

In this study, we clarified that certain soluble factor(s) contained in CM inhibit IFN-γ production from PBMCs by suppressing IL-12 production via inhibiting CD40 expression. We examined the correlation of the expression of CD40/CD40L, IL12, and IFN-γ with lymph node metastasis and prognosis using TCGA cohort data. Although no statistical correlation between the expression of CD40/CD40L, IL12, and IFN-γ and lymph node metastasis, CD40L and IFN-γ were statistically correlated with good prognosis (Figure 7). This finding indicates that the CD40/CD40L–IL-12 axis could be a target for cancer immunotherapy. To reproduce the physiologic immune status in cancer patients, we applied an in vitro simulation model to use the PBMCs isolated from healthy volunteers in the presence of CM derived from oral cancer cell lines. Since PBMCs consist of various types of immune cells, including T cells, B cells, DCs, macrophages, NK cells, and more, we should isolate these cells and analyze their contribution to CM in a future study. Our study could help develop a novel cancer immunotherapy against patients with advanced oral cancer using OK-432.

## 5. Conclusions

**Figure 8:**
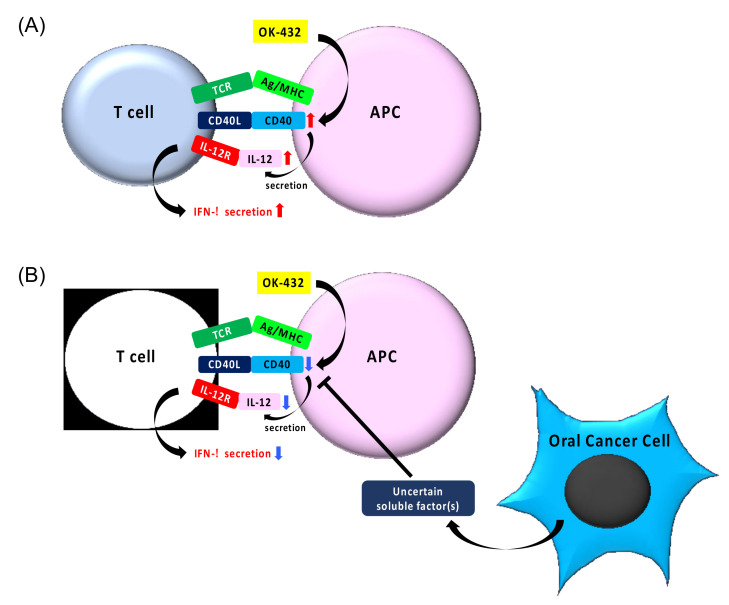
A schematic model of OK-432-induced IFN-γ production via the CD40-IL-12 axis in PBMCs (**A**) and its inhibition by CM derived from oral cancer cells (**B**). OK-432 upregulates CD40 and CD40L expression. Binding of CD40 on antigen-presenting cells and CD40L on activated T cells may induce the expression of IL-12p40, which associates with IL-12p35 to produce IL-12. Secreted IL-12 may induce the production of IFN-γ, and then promotes T cell differentiation and activation. CM including uncertain soluble factor(s) suppresses the CD40/CD40L–IL-12 axis.

Our study suggests that uncertain soluble factor(s) in CM inhibit IFN-γ production in PBMCs by suppressing IL-12 production via inhibiting CD40 expression (Figure 8).

## Data Availability

No new data were created or analyzed in this study. The data presented in this study are available on request from the corresponding author.

## References

[1] Okamoto H., Shoin S., Koshimura S., Shimizu R. (1967). Studies on the anticancer and streptolysin S-forming abilities of hemolytic streptococci. Jpn. J. Microbiol..

[2] Watanabe Y., Iwa T. (1987). Clinical value of immunotherapy with the streptococcal preparation OK-432 in non-small cell lung cancer. J. Biol. Response Mod..

[3] Katano M., Torisu M. (1983). New approach to management of malignant ascites with a streptococcal preparation, OK-432. II. Intraperitoneal inflammatory cell-mediated tumor cell destruction. Surgery.

[4] Uchida A., Micksche M. (1983). Intrapleural administration of OK432 in cancer patients: Activation of NK cells and reduction of suppressor cells. Int. J. Cancer.

[5] Uchida A., Micksche M., Hoshino T. (1984). Intrapleural administration of OK432 in cancer patients: Augmentation of autologous tumor killing activity of tumor-associated large granular lymphocytes. Cancer Immunol. Immunother..

[6] Oshimi K., Kano S., Takaku F., Okumura K. (1980). Augmentation of mouse natural killer cell activity by a streptococcal preparation, OK-432. J. Natl. Cancer Inst..

[7] Oshimi K., Wakasugi H., Seki H., Kano S. (1980). Streptococcal preparation OK-432 augments cytotoxic activity against an erythroleukemic cell line in humans. Cancer Immunol. Immunother..

[8] Matsubara S., Suzuki F., Ishida N. (1979). Induction of immune interferon in mice treated with a bacterial immunopotentiator, OK-432. Cancer Immunol. Immunother..

[9] Sato M., Hayashi Y., Yashida H., Yanagawa T., Yura Y., Urata M., Furumoto M. (1986). Effect of immunotherapy with a streptococcal preparation, OK-432, on the peripheral killer lymphocyte population in patients with head and neck cancer. Immunopharmacological Aspects of OK-432 in Humans.

[10] Kaji R., Yoshida H., Yanagawa T., Sato M. (1989). Monoclonal antibody to a human salivary gland adenocarcinoma cell line: Augmentation of antibody-dependent cell-mediated cytotoxicity activity by streptococcal preparation OK-432 in human salivary gland adenocarcinoma-bearing nude mice given the antibody. J. Biol. Response Mod..

[11] Nakahara S., Tsunoda T., Baba T., Asabe S., Tahara H. (2003). Dendritic cells stimulated with a bacterial product, OK-432, efficiently induce cytotoxic T lymphocytes specific to tumor rejection peptide. Cancer Res..

[12] Sato M., Yoshida H., Yanagawa T., Yura Y., Urata M., Atsumi M., Hayashi Y., Takegawa Y. (1984). Effects of intradermal administration of streptococcal preparation OK-432 on interferon and natural killer cell activities in patients with oral cancer. Int. J. Oral. Surg..

[13] Sato M., Harada K., Yoshida H., Yura Y., Azuma M., Iga H., Bando T., Kawamata H., Takegawa Y. (1997). Therapy for oral squamous cell carcinoma by tegafur and streptococcal agent OK-432 in combination with radiotherapy: Association of the therapeutic effect with differentiation and apoptosis in the cancer cells. Apoptosis.

[14] Clark E.A. (1990). CD40: A cytokine receptor in search of a ligand. Tissue Antigens..

[15] Van Kooten C., Banchereau J. (2000). CD40-CD40 ligand. J. Leukoc. Biol..

[16] Kobayashi M., Fitz L., Ryan M., Hewick R.M., Clarck S.C., Chan S., Loudon R., Sherman F., Perussia B., Trinchieri G. (1989). Identification and purification of natural killer cell stimulatory factor (NKSF), a cytokine with multiple biologic effects on human lymphocytes. J. Exp. Med..

[17] Chan S.H., Perussia B., Gupta J.W., Kobayashi M., Pospisili M., Young H.A., Wolf S.F., Young D., Clark S.C., Trinchieri G. (1991). Induction of interferon-γ production by natural killer cell stimulatory factor: Characterization of the responder cells and synergy with other inducers. J. Exp. Med..

[18] Chan S.H., Kobayashi M., Santoli D., Perussia B., Trinchieri G. (1992). Mechanisms of IFN-γ induction by natural killer cell stimulatory factor (NKSF/IL-12). Role of transcription and mRNA stability in the synergistic interaction between NKSF and IL-2. J. Immunol..

[19] Wolf S.F., Temple P.A., Kobayashi M., Young D., Dicig M., Lowe L., Dzialo R., Fitz L., Ferenz C., Hewick R.M. (1991). Cloning of cDNA for natural killer cell stimulatory factor, a heterodimeric cytokine with multiple biologic effects on T and natural killer cells. J. Immunol..

[20] Gubler U., Chua A.O., Schoenhaut D.S., Dwyer C.M., McComas W., Motyka R., Navabi N., Wolitzky A.G., Quinn P.M., Familletti P.C. (1991). Coexpression of two distinct genes is required to generate secreted bioactive cytotoxic lymphocyte maturation factor. Proc. Natl. Acad. Sci. USA.

[21] Trinchieri G. (1998). Interleukin-12: A cytokine at the interface of inflammation and immunity. Adv. Immunol..

[22] Wang T., Niu G., Kortylewski M., Burdelya L., Shain K., Zhang S., Bhattacharya R., Gabrilovich D., Heller R., Coppola D. (2004). Regulation of the innate and adaptive immune responses by Stat-3 signaling in tumor cells. Nat. Med..

[23] Schroder K., Hertzog P.J., Ravasi T., Hume D.A. (2004). Interferon-gamma: An overview of signals, mechanisms and functions. J. Leukoc. Biol..

[24] Okamoto M., Oshikawa T., Tano T., Ohe G., Furuichi S., Nishikawa H., Ahmed S.U., Akashi S., Miyake K., Takeuchi O. (2003). Involvement of Toll-like receptor 4 signaling in interferon-gamma production and antitumor effect by streptococcal agent OK-432. J. Natl. Cancer Inst..

[25] Ohe G., Sasai A., Uchida D., Tamatani T., Nagai H., Miyamoto Y. (2013). Effect of soluble factors derived from oral cancer cells on the production of interferon-gamma from peripheral blood mononuclear cells following stimulation with OK-432. Oncol. Rep..

[26] Uchida D., Begum N.M., Almofti A., Nakashiro K., Kawamata H., Tateishi Y., Hamakawa H., Yoshida H., Sato M. (2003). Possible role of stromal-cell-derived factor-1/CXCR4 signaling on lymph node metastasis of oral squamous cell carcinoma. Exp. Cell Res..

[27] Kawamata H., Nakashiro K., Uchida D., Harada K., Yoshida H., Sato M. (1997). Possible contribution of active MMP2 to lymph-node metastasis and secreted cathepsin L to bone invasion of newly established human oral-squamous-cancer cell lines. Int. J. Cancer.

[28] Yanagawa T., Hayashi Y., Yoshida H., Yura Y., Nagamine S., Bando T., Sato M. (1986). An adenoid squamous carcinoma-forming cell line established from an oral keratinizing squamous cell carcinoma expressing carcinoembryonic antigen. Am. J. Pathol..

[29] Boyüm A. (1968). Isolation of mononuclear cells and granulocytes from human blood. Isolation of monouclear cells by one centrifugation, and of granulocytes by combining centrifugation and sedimentation at 1 g. Scand. J. Clin. Lab. Invest. Suppl..

[30] Ma X., D’Andrea A., Kubin M., Aste-Amezaga M., Sartori A., Monteiro J., Showe L., Wysocka M., Trinchieri G. (1995). Production of interleukin-12. Res. Immunol..

[31] Kato T., Hakamada R., Yamane H., Nariuchi H. (1996). Induction of IL-12 p40 messenger RNA expression and IL-12 production of macrophages via CD40-CD40 ligand interaction. J. Immunol..

[32] Nemunaitis J., Fong T., Robbins J.M., Edelman G., Edwards W., Paulson R.S., Bruce J., Ognoskie N., Wynne D., Pike M. (1999). Phase I trial of interferon-gamma (IFN-gamma) retroviral vector administered intratumorally to patients with metastatic melanoma. Cancer Gene Ther..

[33] Nasu Y., Bangma C.H., Hull G.W., Lee H.M., Hu J., Wang J., McCurdy M.A., Shimura S., Yang G., Timme T.L. (1999). Adenovirus-mediated interleukin-12 gene therapy for prostate cancer: Suppression of orthotopic tumor growth and pre-established lung metastases in an orthotopic model. Gene Ther..

[34] Saika T., Kusaka N., Mouraviev V., Satoh T., Kumon H., Timme T.L., Thompson T.C. (2006). Therapeutic effects of adoptive splenocyte transfer following in situ AdIL-12 gene therapy in a mouse prostate cancer model. Cancer Gene Ther..

[35] Shurin M.R., Yurkovetsky Z.R., Tourkova I.L., Balkir L., Shurin G.V. (2002). Inhibition of CD40 expression and CD40-mediated dendritic cell function by tumor-derived IL-10. Int. J. Cancer.

[36] Mendez-Samperio P., Garcia E., Vazquez A., Palma J. (2002). Regulation of interleukin-8 by interleukin-10 and transforming growth factor beta in human monocytes infected with mycobacterium bovis. Clin. Diagn. Lab. Immunol..

[37] Banchereau J., Bazan F., Blanchard D., Briere F., Galizzi J.P., van Kooten C., Liu Y.J., Rousset F., Saeland S. (1994). The CD40 antigen and its ligand. Annu. Rev. Immunol..

[38] Zhou Z.H., Wang J.F., Wang Y.D., Qiu Y.H., Pan J.Z., Xie W., Jiang L.Y., Klein B., Zhang X.G. (1999). An agonist anti-human CD40 monoclonal antibody that induces dendritic cell formation and maturation and inhibits proliferation of a myeloma cell line. Hybridoma.

[39] Mauri C., Mars L.T., Londei M. (2000). Therapeutic activity of agonistic monoclonal antibodies against CD40 in a chronic autoimmune inflammatory process. Nat. Med..

